# Effects of magnesium supplementation on muscle soreness in different type of physical activities: a systematic review

**DOI:** 10.1186/s12967-024-05434-x

**Published:** 2024-07-05

**Authors:** Maria Grazia Tarsitano, Federico Quinzi, Katia Folino, Francesca Greco, Francesco Pio Oranges, Claudia Cerulli, Gian Pietro Emerenziani

**Affiliations:** 1 Department of Medical and Surgical Sciences, University Magna Graecia of Catanzaro, 88100 Catanzaro, CZ Italy; 2 Department of Experimental and Clinical Medicine, University “Magna Græcia” of Catanzaro, Catanzaro, CZ Italy; 3 Department of Movement, Human and Health Sciences, University “Foro Italico” of Rome, RM, Italy

**Keywords:** Micronutrient, Fitness, Physical performance, Physical activity, Mg, Dosage

## Abstract

**Background:**

Magnesium is a micronutrient and an intracellular cation responsible for different biochemical reactions involved in energy production and storage, control of neuronal and vasomotor activity, cardiac excitability, and muscle contraction. Magnesium deficiency may result in impaired physical performance. Moreover, magnesium plays an important role on delayed onset muscle soreness after training. Thus, physically active individuals and sport specialists have to pay attention to magnesium supplementation (MgS). However, the type, timing and dosage of magnesium intake are not well elucidated yet. Hence, we aimed to systematically review the literature regarding the effects of MgS on muscle soreness in physically active individuals. We focused exclusively on MgS, excluding those studies in which magnesium was administered together with other substances.

**Methods:**

Three electronic databases and literature sources (PUBMED, SCOPUS and Web of Sciences-Core Collection) were searched, in accordance with PRISMA guidelines. After the database search, 1254 articles were identified, and after excluding duplicates, 960 articles remained. Among these, 955 were excluded following the title and abstract screening. The remaining 5 articles were screened in full text and 4 study met the eligibility criteria.

**Results:**

These studies showed that MgS reduced muscle soreness, improved performance, recovery and induced a protective effect on muscle damage.

**Conclusion:**

To reach these positive effects, individuals engaged in intense exercise should have a Mg requirement 10–20% higher than sedentary people, to be taken in capsules and 2 h before training. Moreover, it is suggested to maintain magnesium levels in the recommended range during the off-season.

**Systematic review registration:**

*PROSPERO registration number*: CRD42024501822.

**Supplementary Information:**

The online version contains supplementary material available at 10.1186/s12967-024-05434-x.

## Introduction

Magnesium (Mg) is an intracellular cation, and it is the fourth most abundant mineral in the human body [1]. Approximately 50% of Mg is stored in bones and the remaining 50% inside cells and organs, while less than 1% is found in the bloodstream [1]. Mg can be transported in the bloodstream bound to proteins, complexed with anions like phosphate bicarbonate and citrate or sulphate and could be ionized. The latter type has been recognized to possess the greatest biological activity [2].

Intestine, bones, and kidneys are crucial for the Mg homeostasis [3]. Specifically, about 30–50% of Mg uptake takes place in the distal small intestine and in the colon, bone tissue is the largest Mg storage system of the body while kidneys are responsible for Mg excretion [3]. Mg absorption depends also on the plasma level of vitamin D. Indeed, high levels of vitamin D enhances Mg absorption and, on the contrary, high consumption of Mg leads to a deficiency or insufficiency of vitamin D [4]. Mg has a fundamental role in the control of neuronal and vasomotor activities, bone formation, cardiac excitability, neuromuscular transmission, muscle contraction and glucose metabolism. Specifically, the calcium transport system that regulates muscle contraction depends on the presence of intracellular Mg [2]. It has been shown that, after physical activity, Mg stored in the extracellular fluid is transferred to bodily tissues where it is urgently needed [5]. The long-term Mg reduction, in the plasma or serum concentration, in parallel with the decrease in the concentration of erythrocyte magnesium, which occurs during or after long-term training, suggests that prolonged exercise may increase Mg requirement [6]. A reduction in Mg concentration (hypomagnesemia) can be observed especially after intense and prolonged exercise whereas brief yet intense bouts of exercise may lead to an increased Mg concentration (hypermagnesemia) [7, 8]. Moreover, Mg plays an important role in glucose metabolism through different mechanisms: glucose homeostasis; regulating phosphorylation; has a fundamental role in many key enzymes [9].

Therefore, a decay in Mg concentration may result in an impaired glucose metabolism [10]. In addition, during exercise, hypomagnesemia leads to glucose depletion, determining a further decline in performance with increased lactate accumulation and increased muscle soreness, an entity of ultrastructural muscle damage that occurs after exercise [11–13]

The Mg increases glucose and piruvate levels in blood, muscles, and brain, decreasing and delaying the accumulation of lactate concentrations in blood and muscles during exercise [14, 15]. This role of Mg is pivotal in glucose homeostasis, enhancing recovery and increasing performance [11].

Nevertheless, even if serum Mg levels fall between normal ranges, it could be possible to perceive muscle pain after intense exercise [16] due to an intracellular magnesium deficiency [17].

In addition, timing and dosage of MgS must be identified.

Previous studies focused on MgS in obesity [18], type 2 diabetes [9, 19], movement disorders [20, 21] and Duchenne muscular dystrophy [22], but few studies on the effect of magnesium on muscle soreness were found. Indeed, in literature, different Mg formulas are adopted: Mg oxide [23–26], Mg-creatine [27], Mg lactate dehydrate [28] Mg citrate [29] or MgS as a cream (MagProTM) [30]. Similarly, different dosages of magnesium administration or combined supplements were used [31–33]. Lastly, the effects of Mg on exercise performance have been extensively studied, however its effects on muscle soreness after exercise in physically active individuals are not well elucidated [10]. Thus, the purpose of this systematic review is to synthesize the effects of MgS on muscle soreness in physically active individuals, focusing on the MgS without supplementation of other substances. Moreover, we summarize the magnesium type, dosage, and duration of the supplementation to reduce muscle soreness.

## Materials and methods

The review was conducted in accordance with the Preferred Reporting Items for Systematic Review and Meta-Analysis (PRISMA 2020) [34] statement and preregistered with PROSPERO (CRD42024501822).

### Search strategy

A systematic search of three electronic databases (Pubmed, Scopus, Web of Science/Core Collection) was performed using predefined search terms deduced from eligibility criteria on the 8th January 2024. The reference lists of identified reviews and included articles were hand searched for potentially relevant articles. The search was conducted using specific keywords (Table 1).

**Table 1:** Databases used to research

Database	Query	8.1.2024
Pubmed	((magnesium) AND ((((((muscle cramps) OR (muscle contraction)) OR (muscle soreness)) OR (doms)) OR (muscle performance)) OR (muscle recovery))) AND (((((((physical activity) OR (post training)) OR (sports)) OR (training)) OR (athletes)) OR (physical exercise)) OR (performance)) AND (2000:2023[pdat])	395
Scopus	( TITLE-ABS-KEY ( magnesium) AND TITLE-ABS-KEY ( muscle AND cramps) OR TITLE-ABS-KEY ( muscle AND contraction) OR TITLE-ABS-KEY ( muscle AND soreness) OR TITLE-ABS-KEY ( doms) OR TITLE-ABS-KEY ( muscle AND recovery) OR TITLE-ABS-KEY ( muscle AND performance) AND TITLE-ABS-KEY ( physical AND activity) OR TITLE-ABS-KEY ( post AND training) OR TITLE-ABS-KEY ( sports) OR TITLE-ABS-KEY ( athletes) OR TITLE-ABS-KEY ( physical AND exercise) OR TITLE-ABS-KEY ( training) OR TITLE-ABS-KEY ( performance)) AND PUBYEAR > 1999 AND PUBYEAR < 2024	548
Web of science (core collection)	((magnesium) AND ((((((muscle cramps) OR (muscle contraction)) OR (muscle soreness)) OR (doms)) OR (muscle performance)) OR (muscle recovery))) AND (((((((physical activity) OR (post training)) OR (sports)) OR (training)) OR (athletes)) OR (physical exercise)) OR (performance)) AND (2000:2023[pdat])	311

### Study selection and data extraction

Following the initial data search, two authors of this study (KF and FPO) independently evaluated the titles and abstracts of all the articles previously identified using the search strategy and screened them for eligibility according to predefined inclusion and exclusion criteria. In case of hesitancy about the inclusion of a study, the two independent reviewers discussed the merits of selection. If a consensus could not be reached, a third reviewer (MGT) was consulted to resolve the issue and an agreement was achieved. After this initial data search, the included studies were read in full and the article data was extracted using a customized form, participant characteristics (age and gender), and study characteristics (magnesium intake, type of sport etc.)

### Eligibility criteria

Studies were further analyzed and considered eligible if the following inclusion criteria were met physically active individuals, English language, both genders, magnesium intake, published in the last 23 years. Reports on animals, cells, non-healthy individuals, and intake of combined supplements were excluded. Moreover, systematic reviews and duplicated publications were excluded.

### Quality assessment and risk of *bias* tool

All studies were checked for methodological quality using a checklist to satisfy the present topic. Quality appraisal was performed independently by three authors using the National Heart, Lung, and Blood Institute Quality Assessment Tools (NHLBI-QAT, https://www.nhlbi.nih.gov/health-topics/study-quality-assessment-tools). A total of 14 criteria were scored. For each criteria 1 point was assigned to the study that met the criteria (“yes”) and 0 point if the study did not meet the criteria (“no” or “cannot determine, not applicable, not reported”). All studies achieved scores ≥ 6 points, so the results were not considered substantially biased. The quality scores ranged from 6 to 13 with a mean value of 7.75. The questions on the assessment tool were designed to help reviewers focus on the key concepts for evaluating a study’s internal validity. They are not intended to create a list that is simply tallied up to arrive at a summary judgement of quality. The score of each study is reported in Supplementary Table.

## Results

**Fig. 1:**
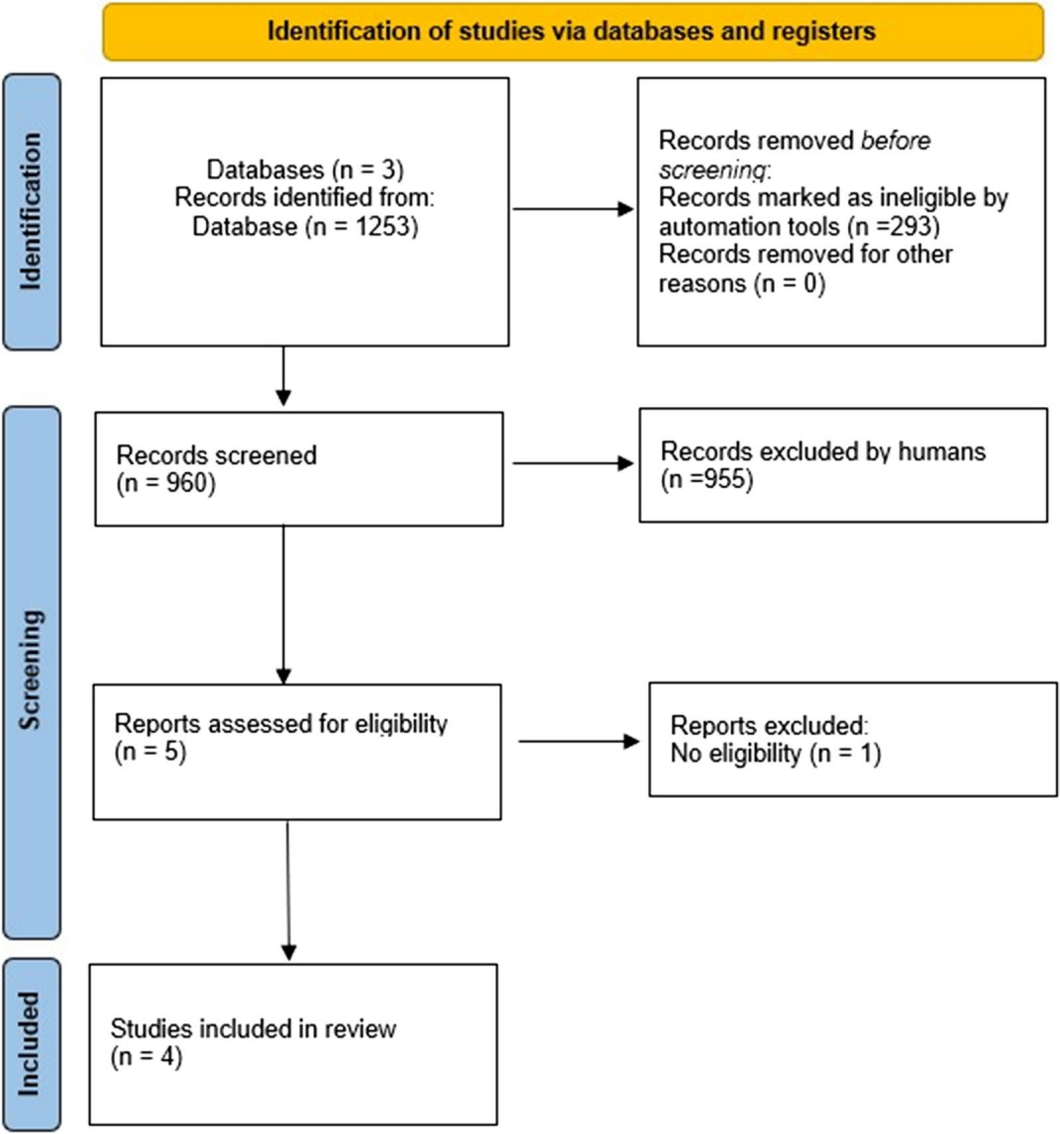
The PRISMA 2020 statement: an updated guideline for reporting systematic reviews

A total of 1253 articles were identified, and after excluding duplicates, 960 articles remained. Among these, 955 were excluded following the title and abstract screening. The remaining 5 articles were screened in full text and 4 study met the eligibility criteria. In particular, the data on the effects of magnesium supplementation on muscle soreness were not present in the fifth paper. For this reason, it was excluded after a detailed review of the article (Fig. 1).

The four eligible studies consisted of 73 participants (60 males and 13 females) between 19–27 years old. One study focused on the effects of MgS on muscle soreness and performance [35], one article focused on running performance [36], and two articles focused on the effects of MgS in athletes involved in team sports [37, 38].

The study of Reno et al. [35] evaluated the effects of MgS on muscle soreness and performance in 9 males and 13 females who completed baseline and post treatment eccentric bench press sessions inducing soreness following performance. MgS consisted in one capsule/die of 350mg of Magnesium’s glycinate. Regarding subjective responses to soreness, a significant positive effect of MgS was observed while no differences were observed in the control group. Post hoc tests showed that soreness ratings were significantly reduced compared to baseline assessment in the MgS group at 24, 36, and 48 h with no significant change in the control group. Moreover, MgS significantly improved feelings of recovery.

Regarding the effects of MgS on long distance running performance, Steward et al. [36] showed that MgS mitigates the exercise-induced stress. Specifically, they investigated the effects of 500 mg of magnesium oxide and stearate in capsule form daily for 7 consecutive days, on nine male recreational runners (27 years old), having a low magnesium diet. During this period, participants engaged in 10 km downhill treadmill run and a running time trial, with a 2-week washout period between trials. The results indicated beneficial effects on blood glucose levels and muscle soreness in the days following the strenuous exercise. Unfortunately, the authors did not report the assessment times.

Regarding MgS in team sports, Cordova Martinez et al. [37] showed that MgS could potentially offer protective effects on muscles through the mitigation of muscle damage parameters in elite athletes. In detail, a cohort of 12 elite basketball players (25.3 years) underwent an intense training regimen consisting of a 2-h morning gym workout and a 3-h afternoon basketball practice. Throughout the competitive season, the athletes received 400 mg/day of MgS. However, the authors did not well specify the type of intake. This supplementation occurred four times during the season at 8-week intervals (T1: October, T2: December, T3: March, T4: April). The study revealed that serum magnesium levels changed muscular damage markers (creatinine, urea, creatine kinase, lactate dehydrogenase, aspartate transaminase, alanine transaminase, aldolase, and total proteins). Specifically, serum magnesium levels were higher at T4 compared to T3, indicating a protective effect of Mg on the muscle damage parameters. Unfortunately, the authors did not report the assessment times.

Finally, Cordova Alfredo et al. [38] explored the effects of MgS in preventing muscle damage in 18 professional cyclists taking part in a 21-day cycling stage race. The cyclists mainly performed aerobic effort with anaerobic peaks in particular moments of the race, like sprinting and climbing. The study concluded that MgS seems to exert a protective effect on muscle damage. In detail, a randomized study design comparing a control group (no supplement) to the MgS group was applied. The effect of oral MgS on serum magnesium level, erythrocyte magnesium levels **(RBC-Mg or er-Mg)**, haematological parameters and inflammation/muscle damage biomarkers (total serum proteins, creatinine, creatin- creatine kinase, lactate dehydrogenase, aspartate transaminase, alanine transaminase and aldolase were determined by standard methods using an autoanalyzer Hitachi 917 (Tokyo, Japan). Myoglobin assessment was performed using a chemiluminescence immunoassay) was evaluated. The results indicated that the recommended dietary allowance (RDA) of MgS allows muscle recovery from intense and strenuous exercise, such as the exercise found in a cycling competition. Moreover, exceeding the MgS’ RDA has a modest effect maintaining muscle integrity. Unfortunately, the authors did not report the assessment times. Details of the selected studies are depicted in Table 2.

**Table 2:** Summary of Magnesium supplementation in physically active individuals

Author(s)	References	Age (years)	Gender	Type of Mg^2+^	Timing	Type of intake	Dosage	Treatment duration	Type of physical activity	Protocol duration	Result
Reno A.M. et al. (2022)	[35]	19–23	M	Glycinate	1 time a day	capsule (n.1)	350 mg	10 days	Brench press	-	Mg significantly reduced muscle soreness and improved perceptual measures linked with performance and recovery
			F		(morning or night)						
Steward consecutive et al. (2019)	[37]	27	M	Oxide/stearate (SG)	-	capsules (n.3)	500 mg	7 days	10 km downhill on treadmill	7 days	Beneficial effects on blood glucose levels and muscle soreness in the days following the strenuous exercise
				Cornflour (CG)							
Cordova M. months et al. (2017)	[37]	PB (25.3)	M	Lactate	1 time a day	-	400 mg	4 times every	Basketball	7	Mg supplementation could potentially offer protective effects on muscle damage parameters in elite athletes
		CG (22.0)	M					8 weeks			
Cordova A. et al. (2019)	[38]	26	M	Pure	1 time a day	capsule (n.1)	400 mg	3 weeks	Professional cyclists	3 week	Mg supplementation seems to exert a protective effect on muscle damage
					(every breakfast)						

*PB* Players Basketball, *CG* Control Group, *SG* Sperimental Group

## Discussion

**Fig. 2:**
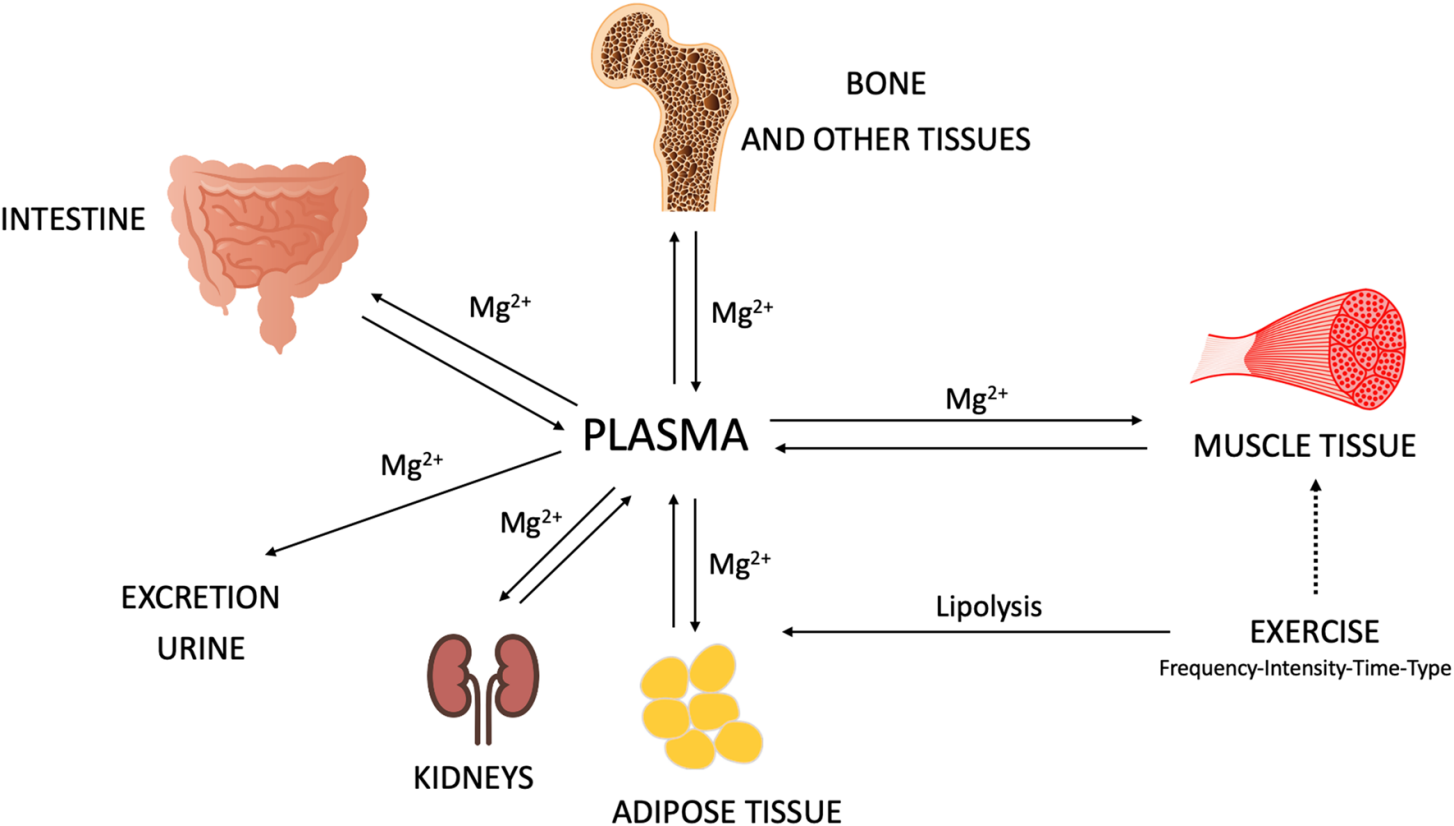
A schematic representation of Mg^2**+**^ fluxes during physical activity. Magnesium exchange and regulation from plasma to different tissues during exercise. Mg^2**+**^**= **Magnesium

The aim of this systematic review is to identify the effects of MgS on muscle soreness in physically active individuals, focusing on the MgS without supplementation of other substances. In addition, we aimed at providing evidence with respect to magnesium type, dosage, and duration of the supplementation. Mg has a fundamental role in the control of neuronal activity, vasomotor activity, bone formation, cardiac excitability, neuromuscular transmission, and muscle contraction [2]. Specifically, both the stimulation and the activity of the calcium transport system in the membranes of the sarcoplasmic reticulum depend on the presence of Mg [2]. Symptomatic magnesium deficiency due to low dietary intake in healthy people is rare, because the kidney limits urinary excretion of this mineral. However, certain health conditions, chronic alcoholism, and/or the use of certain medications can lead to a deficiency of dietary magnesium [39]. During exercise, compartmental shifts of Mg to related extracellular magnesium, were observed. A schematic representation of Mg^2**+**^ flows during physical activity is represented in Fig. 2.

Specifically, high-intensity exercise produces a relative hypermagnesemia while submaximal exercise produces hypomagnesemia. In particular, muscle activities decrease intracellular and plasma Mg concentration. However, the level of Mg in plasma compartment is regulated by the activities of different organs like bone tissue and kidney as reported in Fig. 2 [6]. Physical activity generates Mg depletion [6], this hypomagnesemia leads to further glucose depletion caused by exercise [7, 8]. Then, a further decline in performance occurs with increased lactate accumulation and increased muscle soreness [11]. The concentration of plasma Mg, the principal parameter to evaluate the nutritional status of this mineral, increases after short-lasting high-intensity exercises and decreases after long-lasting exercises [6, 40, 41]. Specifically, during exhausting exercise, a decreased Mg level could inhibit calcium release from the sarcoplasmic reticulum leading to muscle soreness [42]. Moreover, long duration exercise may cause hypomagnesemia and hypoglycemia consequently [14]. Thus, exercise may increase magnesium demand and/or magnesium depletion, leading to magnesium deficiency and to an increase in circulating markers of muscle damage. Magnesium deficiency has been demonstrated to compromise performance and amplify the negative effects of strenuous exercise. Therefore, magnesium supplementation or higher levels of dietary magnesium intake could be useful for physically active individuals with a low or deficient magnesium status [43]. Therefore, hypomagnesemia may also compromise the recovery after training [6, 27, 44, 45]. Some studies found that sustained moderate physical exercise and short-term high intensity exercise increased serum magnesium concentration. Instead of decreased plasma volume, muscle breakdown was suggested as the cause of increased serum magnesium found shortly after exercise [6]. At the same time, the intracellular depletion of magnesium appears impairing the Mg-ATP complex, necessary for the activity of all glycolytic enzymes, protein kinases, and, more generally, all enzymes associated with ATP and phosphate transfer, essential for the muscle contraction [46]. Athletes often do not follow a diet that contains adequate amounts of minerals, including magnesium, which leads to marginal nutrient deficiency and results in substandard training and reduced performance [6]. Therefore, individuals who regularly practice intense exercise should consider increasing their Mg intake by 10–20% more compared to gender-matched sedentary peers. Therefore, during the sports season MgS is strongly recommended [17]. Regarding females, a fluctuation in sex hormones (estrogen and progesterone) influences Mg availability [47] and regulates protein metabolism and muscle recovery processes affecting training [48]. Specifically, different studies show low Mg concentrations during the follicular phase, with an increase during the luteal phase. Thus, during the follicular phase, a higher magnesium intake could be necessary [5, 47].

In addition, there are notable differences between sensitivity and intensity of pain perception between men and women. Women consistently feel pain (perception, description and expression of pain, the use of coping strategies, and the benefit of different treatments) different compared to men. There are convincing findings that biological differences contribute to the observed gender differences. Genetic factors, as well as hormonal factors, act as gender-specific pain mediators [49]. Unfortunately, in our selected study approximately 80% of individuals are men. Thus, we could not perform a gender difference in this issue.

The information regarding the type, timing, and dosage of magnesium intake are poor. Wang et al. [50] speculated that the failure to achieve an optimal magnesium blood levels in athletes and physically active individuals, could be due to wrong dosage or to inappropriate supplementation formulas [50]. In addition to insufficient intake, the bioavailability of different supplementation type may also have masked an effect of MgS on muscle activity. In this systematic review, the studies administered a capsule for MgS with different type of magnesium, including citrate, lactate, and oxide. Ates et al. [51] showed that magnesium citrate is the best type for muscle efficiency. Regarding the dosage of MgS, the studies selected for this systematic review administered from 300 to 500 mg. Generally, the daily magnesium supplementation should be between 360 to 420 mg in adulthood [52]. Thus, individuals who regularly practice intense exercise should increase their MgS by 10–20% compared to an age and gender-matched sedentary person [17]. Therefore, among the studies selected in our systematic review, only one [36] used a higher dosage compared to the suggested supplementation dosage. Reno et al., [35] did not reach the suggested dosage, while the last two studies [37, 38] used a dosage suggested for the general population. Therefore, future studies are needed to assess the effects of high MgS dosage in physically active individuals. In summary, according to the result of this systematic review, the following indications for MgS intake to reduce muscle soreness can be provided: once a day, two hours before training, and mainly during the follicular phase of the menstrual cycle in women [53], maintaining a diet-only magnesium intake during the off-season [2, 17]. In Reno’s et al. [35] study Mg was taken in the morning or at night for 10 days, in Cordova Martinez’ et al. [37] study Mg was taken once a day for 8 weeks without timing detail, lastly in Cordova Alfredo’s et al. [38] study Mg was taken every morning at breakfast for 3 weeks. Since it is known that in humans Mg absorption starts approximately 1 h after oral intake, reaches a plateau after 2–2.5 h up to 4–5 h and then declines [54], our selected studies did not follow the specific suggestion about the timing according to the physical activity. Moreover, they did not give information regarding how many hours before meals or physical activity the MgS was taken.

This systematic review highlights the positive effects of MgS in reducing muscle soreness [35, 36] improving performance, recovery [35] and inducing a protective effect on muscle damage [37, 38]. It has well known that different tested formulation reduced interference of DOMS on daily activities, demonstrating its improvement on a functional aspect of recover. However, the primary of our review was to evaluate the effect of the only MgS [55].

In conclusion, few studies investigated the isolated MgS to reduce soreness and improve muscle performance and recovery. During strenuous exercise, a decrease in Mg level could inhibit calcium release from the sarcoplasmic reticulum, causing muscle soreness. High levels of Mg reduce muscle soreness, useful to improve recovery and training. Therefore, physically active individuals need more Mg than the recommended dose by 10–20%, in capsules and 2 h before physical activity. However, it is suggested to maintain magnesium levels in the recommended range during the off-season. Future studies, with higher sample size, are necessary to clarify the correct type, timing, and dosage of MgS. Moreover, it would be interesting to analyze the effects on MgS on muscles in different diseases like Duchenne muscular dystrophy (Table 3).

**Table 3:** Summary of the indications in a magnesium supplement

Type	Dosage	Type of intake	Timing	On season	Off season
Citrate	10–20% of recommended dietary allowance (RDA)	Capsule	2 h before physical activity	10–20% of RDA	Men: 400–420 mg Women: 310–320 mg (RDA)

### Supplementary Information

Additional file 1.

## Data Availability

Not applicable.
