# Supplementary material for: Effects of magnesium supplementation on muscle soreness in different type of physical activities: a systematic review
Source: J Transl Med. 2024 Jul 5;22:629. doi: 10.1186/s12967-024-05434-x (PMC11227245; doi:10.1186/s12967-024-05434-x)
Supplement: Supplementary file 1 — Additional file 1. [file 12967_2024_5434_MOESM1_ESM.docx]

CORDOVA MARTINEZ, 2017

| **Criteria** | **Yes** | **No** | **Other (CD, NR, NA)*** |
| --- | --- | --- | --- |
| 1. Was the study described as randomized, a randomized trial, a randomized clinical trial, or an RCT? |  | no |  |
| 2. Was the method of randomization adequate (i.e., use of randomly generated assignment)? |  | no |  |
| 3. Was the treatment allocation concealed (so that assignments could not be predicted)? |  |  | cd |
| 4. Were study participants and providers blinded to treatment group assignment? |  |  | cd |
| 5. Were the people assessing the outcomes blinded to the participants' group assignments? |  |  | cd |
| 6. Were the groups similar at baseline on important characteristics that could affect outcomes (e.g., demographics, risk factors, co-morbid conditions)? | yes |  |  |
| 7. Was the overall drop-out rate from the study at endpoint 20% or lower of the number allocated to treatment? |  |  | cd |
| 8. Was the differential drop-out rate (between treatment groups) at endpoint 15 percentage points or lower? |  |  | cd |
| 9. Was there high adherence to the intervention protocols for each treatment group? | yes |  |  |
| 10. Were other interventions avoided or similar in the groups (e.g., similar background treatments)? | yes |  |  |
| 11. Were outcomes assessed using valid and reliable measures, implemented consistently across all study participants? | yes |  |  |
| 12. Did the authors report that the sample size was sufficiently large to be able to detect a difference in the main outcome between groups with at least 80% power? |  |  | nr |
| 13. Were outcomes reported or subgroups analyzed prespecified (i.e., identified before analyses were conducted)? | yes |  |  |
| 14. Were all randomized participants analyzed in the group to which they were originally assigned, i.e., did they use an intention-to-treat analysis? | yes |  |  |

Córdova Martínez A, Fernández-Lázaro D, Mielgo-Ayuso J, Seco Calvo J, Caballero García A. Effect of magnesium supplementation on muscular damage markers in basketball players during a full season. Magnes Res. 2017 May 1;30(2):61-70. doi: 10.1684/mrh.2017.0424. PMID: 28816171.

STEWARD, 2019

| **Criteria** | **Yes** | **No** | **Other (CD, NR, NA)*** |
| --- | --- | --- | --- |
| 1. Was the study described as randomized, a randomized trial, a randomized clinical trial, or an RCT? | yes |  |  |
| 2. Was the method of randomization adequate (i.e., use of randomly generated assignment)? | yes |  |  |
| 3. Was the treatment allocation concealed (so that assignments could not be predicted)? | yes |  |  |
| 4. Were study participants and providers blinded to treatment group assignment? | yes |  |  |
| 5. Were the people assessing the outcomes blinded to the participants' group assignments? | yes |  |  |
| 6. Were the groups similar at baseline on important characteristics that could affect outcomes (e.g., demographics, risk factors, co-morbid conditions)? | yes |  |  |
| 7. Was the overall drop-out rate from the study at endpoint 20% or lower of the number allocated to treatment? | yes |  |  |
| 8. Was the differential drop-out rate (between treatment groups) at endpoint 15 percentage points or lower? | yes |  |  |
| 9. Was there high adherence to the intervention protocols for each treatment group? | yes |  |  |
| 10. Were other interventions avoided or similar in the groups (e.g., similar background treatments)? | yes |  |  |
| 11. Were outcomes assessed using valid and reliable measures, implemented consistently across all study participants? | yes |  |  |
| 12. Did the authors report that the sample size was sufficiently large to be able to detect a difference in the main outcome between groups with at least 80% power? |  |  | nr |
| 13. Were outcomes reported or subgroups analyzed prespecified (i.e., identified before analyses were conducted)? | yes |  |  |
| 14. Were all randomized participants analyzed in the group to which they were originally assigned, i.e., did they use an intention-to-treat analysis? | yes |  |  |

Steward CJ, Zhou Y, Keane G, Cook MD, Liu Y, Cullen T. One week of magnesium supplementation lowers IL-6, muscle soreness and increases post-exercise blood glucose in response to downhill running. Eur J Appl Physiol. 2019 Dec;119(11-12):2617-2627. doi: 10.1007/s00421-019-04238-y. Epub 2019 Oct 17. PMID: 31624951.

LINDSY KASS, 2015

| **Criteria** | **Yes** | **No** | **Other (CD, NR, NA)*** |
| --- | --- | --- | --- |
| 1. Was the study described as randomized, a randomized trial, a randomized clinical trial, or an RCT? | yes |  |  |
| 2. Was the method of randomization adequate (i.e., use of randomly generated assignment)? | yes |  |  |
| 3. Was the treatment allocation concealed (so that assignments could not be predicted)? | yes |  |  |
| 4. Were study participants and providers blinded to treatment group assignment? | yes |  |  |
| 5. Were the people assessing the outcomes blinded to the participants' group assignments? | yes |  |  |
| 6. Were the groups similar at baseline on important characteristics that could affect outcomes (e.g., demographics, risk factors, co-morbid conditions)? |  | no |  |
| 7. Was the overall drop-out rate from the study at endpoint 20% or lower of the number allocated to treatment? |  |  | nr |
| 8. Was the differential drop-out rate (between treatment groups) at endpoint 15 percentage points or lower? |  |  | nr |
| 9. Was there high adherence to the intervention protocols for each treatment group? |  |  | cd |
| 10. Were other interventions avoided or similar in the groups (e.g., similar background treatments)? |  | no |  |
| 11. Were outcomes assessed using valid and reliable measures, implemented consistently across all study participants? | yes |  |  |
| 12. Did the authors report that the sample size was sufficiently large to be able to detect a difference in the main outcome between groups with at least 80% power? |  |  | nr |
| 13. Were outcomes reported or subgroups analyzed prespecified (i.e., identified before analyses were conducted)? | yes |  |  |
| 14. Were all randomized participants analyzed in the group to which they were originally assigned, i.e., did they use an intention-to-treat analysis? | yes |  |  |

Kass LS, Poeira F. The effect of acute vs chronic magnesium supplementation on exercise and recovery on resistance exercise, blood pressure and total peripheral resistance on normotensive adults. J Int Soc Sports Nutr. 2015 Apr 24;12:19. doi: 10.1186/s12970-015-0081-z. Erratum in: J Int Soc Sports Nutr. 2018 Jul 25;15(1):36. PMID: 25945079; PMCID: PMC4419474.

RENO, 2022

| **Criteria** | **Yes** | **No** | **Other (CD, NR, NA)*** |
| --- | --- | --- | --- |
| 1. Was the study described as randomized, a randomized trial, a randomized clinical trial, or an RCT? |  |  | nr |
| 2. Was the method of randomization adequate (i.e., use of randomly generated assignment)? |  |  | nr |
| 3. Was the treatment allocation concealed (so that assignments could not be predicted)? | yes |  |  |
| 4. Were study participants and providers blinded to treatment group assignment? | yes |  |  |
| 5. Were the people assessing the outcomes blinded to the participants' group assignments? | yes |  |  |
| 6. Were the groups similar at baseline on important characteristics that could affect outcomes (e.g., demographics, risk factors, co-morbid conditions)? | yes |  |  |
| 7. Was the overall drop-out rate from the study at endpoint 20% or lower of the number allocated to treatment? |  |  | nr |
| 8. Was the differential drop-out rate (between treatment groups) at endpoint 15 percentage points or lower? |  |  | nr |
| 9. Was there high adherence to the intervention protocols for each treatment group? | yes |  |  |
| 10. Were other interventions avoided or similar in the groups (e.g., similar background treatments)? |  |  | nr |
| 11. Were outcomes assessed using valid and reliable measures, implemented consistently across all study participants? |  |  | nr |
| 12. Did the authors report that the sample size was sufficiently large to be able to detect a difference in the main outcome between groups with at least 80% power? |  |  | nr |
| 13. Were outcomes reported or subgroups analyzed prespecified (i.e., identified before analyses were conducted)? | yes |  |  |
| 14. Were all randomized participants analyzed in the group to which they were originally assigned, i.e., did they use an intention-to-treat analysis? | yes |  |  |

Reno AM, Green M, Killen LG, O'Neal EK, Pritchett K, Hanson Z. Effects of Magnesium Supplementation on Muscle Soreness and Performance. J Strength Cond Res. 2022 Aug 1;36(8):2198-2203. doi: 10.1519/JSC.0000000000003827. Epub 2020 Oct 1. PMID: 33009349.

CORDOVA A, 2019

| **Criteria** | **Yes** | **No** | **Other (CD, NR, NA)*** |
| --- | --- | --- | --- |
| 1. Was the study described as randomized, a randomized trial, a randomized clinical trial, or an RCT? | yes |  |  |
| 2. Was the method of randomization adequate (i.e., use of randomly generated assignment)? | yes |  |  |
| 3. Was the treatment allocation concealed (so that assignments could not be predicted)? |  |  | nr |
| 4. Were study participants and providers blinded to treatment group assignment? |  |  | nr |
| 5. Were the people assessing the outcomes blinded to the participants' group assignments? |  |  | nr |
| 6. Were the groups similar at baseline on important characteristics that could affect outcomes (e.g., demographics, risk factors, co-morbid conditions)? | yes |  |  |
| 7. Was the overall drop-out rate from the study at endpoint 20% or lower of the number allocated to treatment? |  |  | nr |
| 8. Was the differential drop-out rate (between treatment groups) at endpoint 15 percentage points or lower? |  |  | nr |
| 9. Was there high adherence to the intervention protocols for each treatment group? | yes |  |  |
| 10. Were other interventions avoided or similar in the groups (e.g., similar background treatments)? |  |  | nr |
| 11. Were outcomes assessed using valid and reliable measures, implemented consistently across all study participants? | yes |  |  |
| 12. Did the authors report that the sample size was sufficiently large to be able to detect a difference in the main outcome between groups with at least 80% power? |  |  | nr |
| 13. Were outcomes reported or subgroups analyzed prespecified (i.e., identified before analyses were conducted)? | yes |  |  |
| 14. Were all randomized participants analyzed in the group to which they were originally assigned, i.e., did they use an intention-to-treat analysis? | yes |  |  |

Córdova A, Mielgo-Ayuso J, Roche E, Caballero-García A, Fernandez-Lázaro D. Impact of Magnesium Supplementation in Muscle Damage of Professional Cyclists Competing in a Stage Race. Nutrients. 2019 Aug 16;11(8):1927. doi: 10.3390/nu11081927. PMID: 31426321; PMCID: PMC6723322.
